# Spontaneous muscle hematoma in older patients with COVID-19: two case reports and literature review

**DOI:** 10.1186/s12877-020-01963-4

**Published:** 2020-12-22

**Authors:** Sara Rogani, Valeria Calsolaro, Riccardo Franchi, Alessia Maria Calabrese, Chukwuma Okoye, Fabio Monzani

**Affiliations:** Geriatrics Unit, Department of Clinical and Experimental Medicine, University of Pisa, Via Savi 10, 56126 Pisa, Italy

**Keywords:** COVID-19, Endothelial dysfunction, Vascular disease, Muscle haematoma, Case report

## Abstract

**Background:**

In late December 2019, a cluster of pneumonia cases due to a novel betacoronavirus, SARS-CoV-2 was reported in China. The so-called COVID 19 is responsible not only for respiratory symptoms, from mild up to pneumonia and even acute respiratory distress syndrome, but also for extrapulmonary involvement.

**Cases presentation:**

Here we present two cases of spontaneous muscle hematoma in patients with SARS-CoV-2 infection, both on therapeutic LMWH for atrial fibrillation: the first one was an 86-year-old Caucasian female with a history of hypertensive cardiomyopathy and the second one was an 81-year-old Caucasian male with a history of hypertension, diabetes and ischemic heart disease. Blood tests revealed a considerable drop of hemoglobin and alterations of coagulation system. In both cases, embolization of femoral artery was performed. A few other cases of bleeding manifestations are reported in literature, while a lot has been published about the hypercoagulability related to COVID-19.

**Conclusions:**

Our reports and literature review highlight the need of active surveillance for possible hemorrhagic complications in patients with SARS-CoV-2 infection.

## Background

In late December 2019, a cluster of pneumonia cases of unknown etiology was reported in the city of Wuhan in Hubei Province, China. A novel betacoronavirus, SARS-CoV-2, spread by inter-human transmission, is considered as the etiological agent of this disease [1, 2]. The World Health Organization (WHO) officially named the coronavirus disease “COVID-19” and declared a global pandemic in 2020. The spectrum of clinical manifestations of SARS-Cov-2 infection ranges from fever, fatigue, myalgia, cough and dyspnoea to pneumonia and even acute respiratory distress syndrome. Extrapulmonary involvement, such as gastrointestinal (diarrhea, nausea/vomiting, abdominal pain), cardiovascular (arrhythmias, myocardial injury) and renal (acute renal injury) have been reported as well. There is emerging evidence of the role of vascular disease in COVID-19 and clinical reports increasingly suggest a confluence of endothelial dysfunction, thrombosis and dysregulated inflammation [3]. Here we present two cases of older patients with SARS-Cov-2 infection, who presented spontaneous muscle hematoma on anticoagulation therapy with heparin in therapeutic dose.

## Cases presentation

### Case 1

The first patient was an 86-year-old woman with a history of hypertensive cardiomyopathy with severe mitral regurgitation and atrial fibrillation on anticoagulant therapy with warfarin. She presented to the Emergency Department after experiencing a sudden onset of shortness of breath on mild exertion and peripheral edema.

At admission, the patient was afebrile, eupneic in oxygen therapy at rest. Thoracic physical examination revealed decreased breath sounds and crackles in the lower lung lobes, bilaterally.

Blood investigations revealed a total leucocyte count of 9140/mcL (normal 4000–11,000/mcL), Hb 11 g/dL (normal 13–18 g/dL), C-reactive protein (CRP) 15.8 mg/dL (normal < 0.50 mg/dL), Procalcitonin (PCT) 0.32 ng/L (normal < 0.5 ng/L), fibrinogen 707 mg/dL (normal 200–400 mg/dL), creatinine 1.23 mg/dL (normal 0.7–1.20 mg/dL), INR 2.36 (normal 0.82–1.19). Chest CT revealed bilateral pleural effusion with passive atelectasis of the lower lobes. Patient was diagnosed with COVID-19 on April the 8th, 2020, on the basis of RT-PCR testing that detected SARS-CoV-2. The patient was admitted to our Geriatric department and began to receive hydroxychloroquine, intravenous ceftriaxone and doxycycline, as per local protocol, observing a good clinical response and the decrease of inflammation markers. Weaning and discontinuation of oxygen therapy was possible once the patient was stable with satisfactory oxygen saturation. The anticoagulation (AC) therapy was maintained, however, we decided to switch AC therapy from warfarin to low molecular-weight heparin (LMWH) enoxaparin 6000 U BID.

**Fig. 1:**
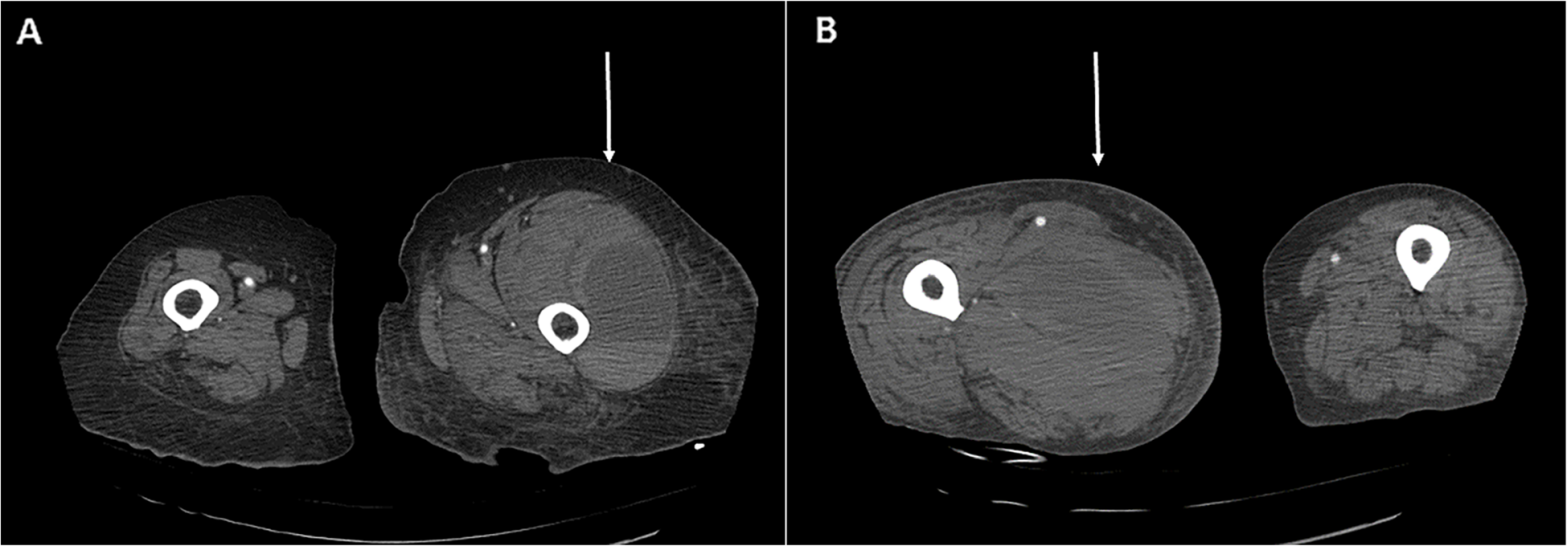
Computed Tomography images of the two patients’ hematomas. This image shows the 11 x 8 x 14 cm hematoma in the Case 1’s left thigh (**a**) and the 12 x 10 x 25 cm Case 2’s hematoma in the right thigh (**b**) (white arrows)

Suddenly, 5 days after admission, she started complaining of pain and loss of movement of her left leg, which appeared swollen. Venous compression ultrasound (CUS) and evaluation of D-dimer excluded deep vein thrombosis, but a Chest computed tomography (CT) scan with contrast revealed an 11 × 8 × 14 cm hematoma in the left thigh (Fig. 1). Laboratory tests showed a fall in hemoglobin from 11 g/dL to 7.8 g/dL and, despite the absence of active arterial extravasation, prophylactic embolization of small branches of superficial and deep femoral artery was performed. Blood tests at that time also showed alterations of coagulation system, with reduced prothrombin time, increased fibrinogen and international normalized ratio (INR), despite a platelet count within normal limits. Interleukin-6 levels were 67,9 pg/mL (normal < 12.7 pg/mL). Overall, the patient was transfused 3 unit of packed blood cells, which raised her hemoglobin to 10.1 g/dL. Table 1 shows the patient’s routine blood test during the hospitalization. In the following days, the patient’s hemoglobin remained stable and the hematoma showed no evidence of further expansion. The patient recovered completely and was discharged shortly after the event.

**Table 1:** Blood investigations of both patients

	Case 1				Case 2			
**Blood tests**	**DAY 1**	**DAY 4**	**DAY 19**	**DAY 23**	**DAY 1**	**DAY 2**	**DAY 10**	**DAY 14**
**RBC IU/mcL**	4.310.000	4.710.000	2.970.000	3.710.000	3.7400.00	3.090.000	3.460.000	2.740.000
**Hb g/dL**	11,0	11,9	7,80	9.9	12,5	10,3	11,10	8,50
**HCT %**	33	38	24,30	30.7	36,6	29,9	34,10	25,2
**MCV fL**	77	80	81,80	82,7	97,9	96,4	98,60	92
**WBC IU/mcL**	9140	8800	13.360	8300	15.640	9390	12.600	17.120
**N %**	83	74	82	77,9	88,5	90,3	91,00	90,7
**L %**	9	17	10	12,5	5,7	3,6	2,90	4,2
**M %**	8	9	7,4	9	5,6	6,1	6	5
**E %**	0	0	0,3	0,5	0,1	0	0,10	0,1
**B %**	0	0	0	0	0,1	0	0,00	0
**PLT IU/mcL**	155.000	167.000	301.000	233.000	327.000	256,000	290.000	211.000
**PT %**	17	66		75,00	36	49	59	
**INR**	4,00	1,34		1,19	2,1	1,67	1,43	
**aPTT sec.**	40,3	30,4		32,4	28,5	23,4	36,70	
**aPTT Ratio**	1,40	1,06		1,13	0,99	0,82	1,27	
**Fibrinogen mg/dL**	707	805		421		350		
**D-dimer mg/L**		0,3		0,47		1,95		
**CRP mg/dL**	15,88	13,04		11,37	16,69	11,41	1,64	
**PCT ng/mL**	0,32	0,32			0,9	0,9		
**BNP pg/mL**	571	945		907	2523	1367	229	
**IL-1 pg/mL**					3,5			
**IL-6 pg/mL**		67,9			6,1			
**IL-10 pg/mL**					0,5			
**TNF-α pg/mL**					1,8			
**MCP-1 pg/mL**		356			228			
**Creatinine mg/dL**	1,23	1,07	1,53	0,81	1,92	1,56	1,82	1,58

*Abbreviations*: *RBC *red blood cells, *Hb *haemoglobin, *HCT *hematocrit, *MCV *mean corpuscular volume, *WBC *white blood cells, *N *neutrophils, *L *lymphocytes, *M *monocytes, *E *eosinophils, *B *basophils, *PLT *platelets, *INR *international normalized ratio, *PT *prothrombin time, *aPTT *activated partial thromboplastin time, *CRP *C-reactive protein, *PCT *procalcitonin, *BNP *brain natriuretic peptide, *IL-1 *interleukin 1, *IL-6 *interleukin 6, *IL-10 *interleukin 10, *TNF-α *tumor necrosis factor α, *MCP-1 *monocyte chemoattractant protein-1

### Case 2

Another patient with similar findings was seen at our Geriatric department. The patient was an 81-year-old man with a history of hypertension, diabetes, ischemic heart disease and dilated cardiomyopathy with reduced left ventricular ejection fraction on echocardiogram, who presented to the Emergency Department with cough and shortness of breath. At admission the patient was oriented, febrile and had a respiratory rate of 15 breaths per minute and oxygen saturation of 97% while he was receiving oxygen therapy (10 L/min). He was tachycardic and ECG detected atrial fibrillation.

Laboratory tests showed a total leucocyte count of 15,640/mcL (N 88.5%, L 5.7%), Hb 12.5 g/dL, C-reactive protein (CRP) 16.69 mg/dL, PCT 0.95 ng/mL, fibrinogen 350 mg/dL, creatinine 1.92 mg/dL, Brain Natriuretic Peptide (BNP) 2523 pg/mL (normal < 100 pg/mL), INR 2.1. CT scan revealed bilateral ground-glass opacities and crazy-paving pattern with thickened interlobular septae and intralobular lines, with bilateral consolidations and thoracic lymphadenopathy. Serological tests detected the presence of serum IgM antibodies against SARS-Cov-2. Once admitted to our Geriatric ward, blood tests were repeated; INR dropped to 1.56 and creatinine values dropped to 1.67 mg/dL (CKD-EPI 41 mL/min/1.73 m^2^), therefore, accounting for the patient’s body weight of 83 Kg, anticoagulation therapy with LMWH (enoxaparin 8000 U BID) was administered. Blood cultures were collected (gram-positive cocci positivity detected), antimicrobial broad-spectrum therapy was started, observing a good clinical response and the decrease of inflammation markers.

**Fig. 2:**
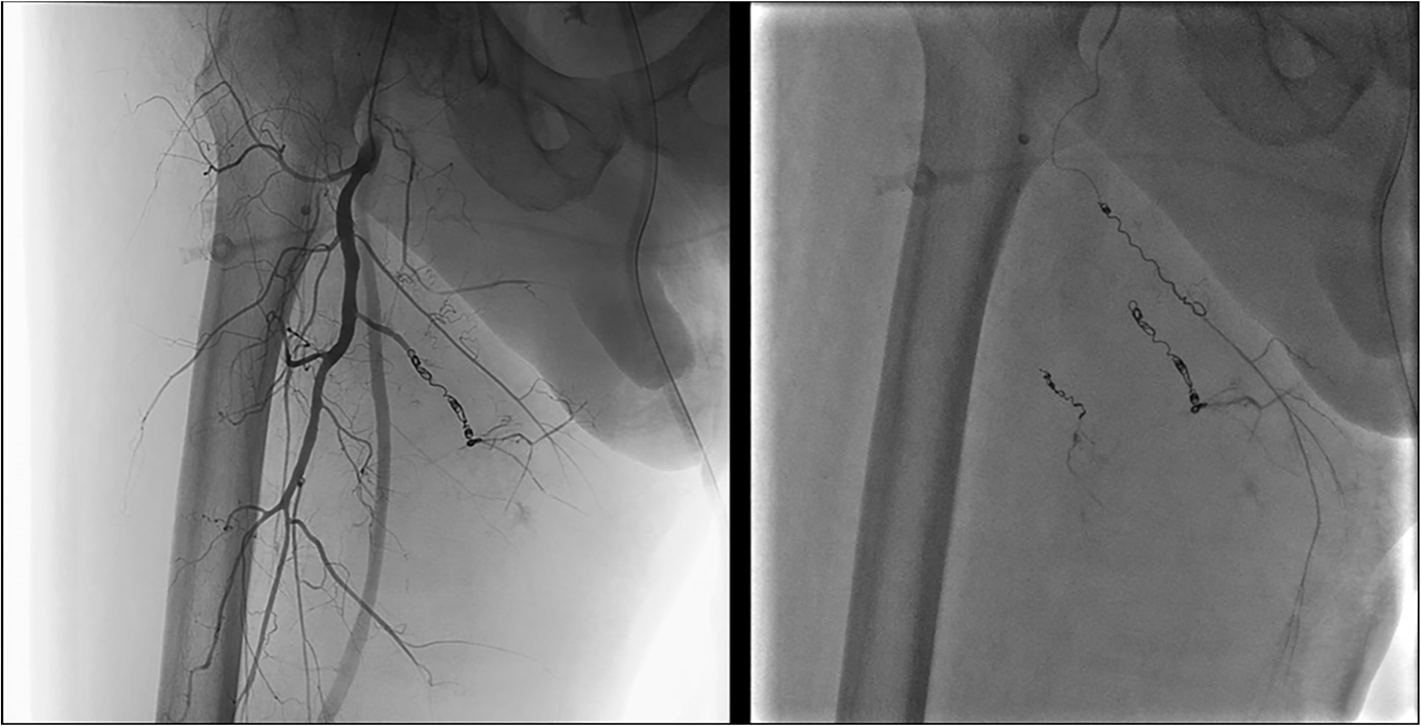
Selective catheterism and coil embolization of hematoma’s arterial afferences. The image shows the catheterized and embolized arterial afferences of Case 2’s hematoma

On the ninth day of admission, similarly to the first case we reported, the patient presented right thigh hematoma causing pain and loss of movement. X-ray showed no sign of hip fracture. Laboratory investigations at the time revealed a drop in hemoglobin from 12,5 g/dL to 6,6 g/dL and showed alterations of coagulation system, with decreased prothrombin time, increased partial thromboplastin time and INR, despite a platelet count within normal limits. A CT scan with contrast detected a 12 × 10 × 25 cm hematoma in the right thigh with four foci of active arterial extravasation (i.e. active bleeding) (Fig. 1). Transcatheter angiography directly identified arterial injury and the source of the bleeding, so that coil and gelfoam embolization of branches of deep femoral artery was performed (Fig. 2). The patient was transfused 2 unit of packed blood cells. Table 1 shows the patient’s routine blood test during the hospitalization. Although the procedure was successful and there was no evidence of further arterial extravasation, the patient died in the following days.

The patients described in the current manuscript have not been reported in any other submission. Written informed consent for publication was obtained from the first patient and from the next of keen of the second one.

## Discussion

There are several cases in the recent literature of different patterns of vascular disease in patients with SARS-CoV-2 infection, most of which point towards the hypercoagulability related to COVID-19.

Melissano et al. reported a high number of cases hospitalized for pneumonia SARS-CoV-2 induced, associated with ischemia of lower and upper limbs and a significant number of deep venous thrombosis among both ventilated and unventilated patients. Most of the patients were already receiving antithrombotic prophylaxis [3]. In a retrospective study performed in two French intensive care units, Llitjos et al. found a high rate of venous thromboembolism in anticoagulated patients with COVID-19-related ARDS, supported with mechanical ventilation[4]. The possible link between thromboembolic events and the respiratory failure in COVID-19 has been widely reported. Gattinoni et al. observed a discrepancy between the well-preserved lung compliance and the severity of hypoxemia in patients with COVID-19 pneumonia. A possible explanation for such severe hypoxemia occurring in compliant lungs, in contrast to expectations for severe ARDS, could be a loss of lung perfusion regulation and hypoxic vasoconstriction [5]. This atypical presentation of ARDS seems to be associated to complement-mediated microvascular injury and thrombosis, as shown in recent autoptic studies [6]. Compared to the growing attention given to hypercoagulability associated to COVID-19, a little has been reported about the hemorrhagic problems in patients with SARS-CoV-2 infection. Even though muscle hematomas have always been recognized as a possible complication of LMWH treatment of older adults [7–9], attention should be driven to possible hemorrhagic complications COVID19-related during LMWH treatment [10].

In a group of 41 Thai patients (4 males and 37 females, age between 7 and 74 years) affected by COVID-19, Joob et al.. reported only one patient presenting with bleeding manifestation (petechiae) and he was firstly misdiagnosed as dengue fever [11]. Guotao et al.. described the case of an 83-year-old man with SARS-CoV-2 infection presenting hematochezia, with no source of bleeding identified by colonoscopy or abdominal CT scan. The authors considered the possibility that manifestations including hematochezia may be secondary to an alternate point of entry of SARS-CoV-2 virus [12]. Zulfiqar et al.. described the case of a 65-year-old woman with COVID-19 presenting with immune thrombocytopenic purpura complicated by subarachnoid microhemorrhage [13]. Sharifi-Razavi et al.. reported the case of a 79-year-old man affected by COVID-19, with a history of fever and cough and acute loss of consciousness; brain CT revealed a massive intracerebral hemorrhage. He was not hypertensive nor on anticoagulant therapy. The authors suggested that brain ACE2 could be involved in SARS-CoV-2 infection and its dysfunction could lead to disruption of vascular autoregulation [14]. Conti et al.. reported two cases of spontaneous ileo-psoas hematomas in hospitalized patients for SARS-CoV-2-related bilateral interstitial pneumonia and supported with C-PAP ventilation. They were a 76-year-old man and a 72-year-old woman, both treated with LMWH but at different dosages: the first one with prophylactic LMWH, the second one with therapeutic dosage because of concomitant deep venous femoral thrombosis. Blood investigations revealed normal PLT, PT and PTT; they both received radiological embolization of the epigastric inferior artery. The cause of the bleeding remained unclear; the authors suggested a possible connection with the presence of cough and the use of C-PAP ventilation other than the emerging disorders of the coagulation system [15]. Recently, Mattioli et al. published a case report of neck and upper chest spontaneous hematoma in an 84-year-old patient prescribed with prophylactic dose of LMWH [16].

Finally, our Interventional Radiology team reported other four cases of spontaneous bleeding in patients with COVID-19, all treated with therapeutic dosage of LMWH: a 74-year-old woman with rupture of superior thoracic artery complicated by left pectoral muscle hematoma, a 71-year-old man with left iliopsoas muscle hematoma, a 71-year-old woman with a rectus sheath hematoma due to both right and left inferior epigastric arteries injury and a 81-year-old man with a large left thigh hematoma. They all underwent coil embolization. The authors suggested a traumatic mechanism underlying the bleeding in two cases (mobilization in the prone position in the first one, electrical cardioversion in the second one) [17].

No evidence of trauma was found in the two cases we described. Although hemorrhagic complications in soft tissues, muscles, retroperitoneum [18, 19], are quite common even in absence of SARS-CoV-2 infection in patients treated with LMWH or anticoagulants, in our reports the temporal sequence suggests that COVID-19 was a contributing factor in the development of spontaneous muscle hematoma.

## Conclusions

In conclusion, emerging data confirm the broad range of COVID-19 complications, above the respiratory ones. Among them, vascular involvement in COVID-19 appears to be related not only to hypercoagulability features, but also to bleeding ones. We recommend caution and active surveillance for possible hemorrhagic complications in patients with SARS-CoV-2 infection, in particular in those treated with LMWH or at higher hemorrhagic risk.

## Data Availability

The presented data are available under request, as anonymized files. To have access to the data, please contact Prof F. Monzani, Director of the Geriatrics Unit (Department of Clinical and Experimental Medicine, University of Pisa), Email: fabio.monzani@med.unipi.it, Phone: +393337733135.

## Abbreviations

COVID: Corona virus disease
CRP: C-reactive protein
PCT: Procalcitonin
CT: Computed tomography
RT-PCR: Real-time polymerase chain reaction
LMWH: Low molecular-weight heparin
CUS: Venous compression ultrasound
INR: International normalized ratio
BNP: Brain natriuretic peptide
ARDS: Acute respiratory distress syndrome
ACE2: Angiotensin-converting enzyme-2
RAS: Reninangiotensin system
RBC: Red blood cells
Hb: Haemoglobin
HCT: Hematocrit
MCV: Mean corpuscular volume
WBC: White blood cells
N: Neutrophils
L: Lymphocytes
M: Monocytes
E: Eosinophils
B: Basophils
PLT: Platelets
PT: Prothrombin time
aPTT: Activated partial thromboplastin time
IL-1: Interleukin 1
IL-6: Interleukin 6
IL-10: Interleukin 10
TNF-α: Tumor necrosis factorα
MCP-1: Monocyte chemoattractant protein-1
